# The eye in cerebral malaria: what can it teach us?

**DOI:** 10.1016/j.trstmh.2008.11.003

**Published:** 2009-07

**Authors:** Richard J. Maude, Arjen M. Dondorp, Abdullah Abu Sayeed, Nicholas P.J. Day, Nicholas J. White, Nicholas A.V. Beare

**Affiliations:** a Faculty of Tropical Medicine, Mahidol University, 420/6 Rajvithi Road, Bangkok 10400, Thailand; b Centre for Clinical Vaccinology and Tropical Medicine, Nuffield Department of Clinical Medicine, John Radcliffe Hospital, Oxford, UK; c Chittagong Medical College Hospital, Chittagong, Bangladesh; d St Paul's Eye Unit, Royal Liverpool University Hospital, Prescot Street, Liverpool L7 8XP, UK

**Keywords:** Cerebral malaria, *Plasmodium falciparum*, Retinopathy, Microcirculatory flow, Sequestration, Pathophysiology, Coma

## Abstract

The pathophysiology of coma in cerebral malaria (CM) is not well understood. Obstruction of microcirculatory flow is thought to play a central role, but other hypotheses include roles for parasite- and host-derived factors such as immune mediators, and for increased blood–brain barrier permeability leading to raised intracranial pressure. The retinal vasculature is a direct extension of the cerebral vasculature. It is the only vascular bed easily accessible for visualisation and provides a unique opportunity to observe vascular pathology and its effect on neurological tissue. A specific retinopathy has been well described in African children with CM and its severity correlates with outcome. This retinopathy has been less well described in adults. The central mechanism causing malarial retinopathy appears to be microvascular obstruction, which has been demonstrated in affected retinas by fluorescein angiography. The presence in a central nervous system tissue of microvascular obstruction strongly supports the hypothesis that the sequestration of erythrocytes in small blood vessels and consequent obstruction of microcirculatory flow is an important mechanism causing coma and death in CM. Despite advances in the antimalarial treatment of severe malaria, its mortality remains approximately 15–20%. Adjunctive treatment targeting sequestration is a promising strategy to further lower mortality.

## 1 Introduction

The retinopathy of severe malaria has been well described in African children, and its prognostic and diagnostic value is established in these patients.^1^ Its full assessment requires specialist techniques and training that will not be generally available in most tropical countries in the foreseeable future. It is also an important tool to further our understanding of how malaria causes morbidity and death. This role as a ‘window into the brain’ in severe malaria is one that is just beginning to be realised, but already many pathophysiological insights have been gained. With the further application of established ocular imaging techniques to children and adults with severe malaria as well as the development of new methods of imaging this role is likely to expand.

## 2 Clinical utility

The severity of malarial retinopathy correlates with mortality and duration of coma in African children with cerebral malaria (CM), suggesting that the retinopathy is related to the pathophysiology of the disease and is not an epiphenomenon.^1^ Approximately two-thirds of such patients have retinopathy that can be seen using an indirect ophthalmoscope,^1^ and the mortality in this group is more than double that of comatose children with no retinopathy.^2^ Approximately one-half of patients with severe malarial anaemia have retinopathy, but it is usually much milder than that seen in CM.^1^ Retinopathy is rarely seen in patients with uncomplicated malaria.^3^

The retinopathy of severe malaria has four main components: retinal whitening; vessel discolouration; haemorrhages; and papilloedema.^4^ Cotton wool spots are also seen and are distinct from retinal whitening. The vessel changes and pattern of retinal whitening appear to be unique to this disease.^3^, ^4^ Whilst papilloedema and retinal haemorrhages can be visualised with an ordinary direct ophthalmoscope by a non-expert, the distinctive retinal whitening and vessel abnormalities are found mostly in the peripheral retina and thus require indirect ophthalmoscopy.^4^ With training in indirect ophthalmoscopy, however, 95% of malaria-related retinal changes that are detected by an ophthalmologist may be observed by a non-ophthalmologist (T.E. Taylor, unpublished data). The interobserver concordance between ophthalmologists for grading the severity of malarial retinopathy has also proved satisfactory.^5^

Detection of retinopathy of severe malaria has been proposed as a bedside test to distinguish African children with CM from those with coma due to other causes.^4^ This was supported by a large, prospective autopsy study of children with fatal CM in Malawi where it was found that malarial retinopathy was better than any other clinical or laboratory feature in distinguishing malarial from non-malarial coma.^6^ The clinical presentation of paediatric severe malaria with reduced consciousness is not specific. Up to one-third of comatose, malaria film-positive patients in high transmission areas of the tropics have a non-malarial cause for their coma.^7^ In high transmission areas, the high background prevalence of peripheral blood parasitaemia makes the diagnosis of CM more difficult, whereas in low transmission settings the combination of coma and positive malaria blood film is highly specific.

## 3 Pathophysiological mechanisms

There is good evidence that the pathological mechanisms which produce the retinopathy seen in severe malaria are the same as those which cause coma in CM. As well as correlating with disease severity, the retinal changes have histological correlates that match histological findings in the brain. Sequestration of infected erythrocytes occurs in the retinal microvasculature in the same fashion as the cerebral microvasculature and is thought to cause the retinal vessel discolouration.^8^ The number of retinal haemorrhages correlates with the density of brain haemorrhages and, like cerebral haemorrhages, they have fibrin at their centre.^9^ This is not surprising as the retina is embryologically derived from the same neuroectoderm as the brain and has the same type of vasculature within a structure of neurons and glial cells.

Reduced blood flow is the pathological sequel of microcirculatory obstruction. This is suggested by the histological appearance of cytoadherent erythrocytes containing mature forms of the parasite sequestered in the microvasculature, narrowing vessel lumens. The physical obstruction by these rigid cytoadherent parasitised erythrocytes is compounded by reduced red cell deformability and adhesive forces between infected erythrocytes (autoagglutination) and between infected and uninfected erythrocytes (rosetting).^10^ Impaired perfusion has been demonstrated in vivo by fluorescein angiography of the retina in 28 of 34 children with CM in a study in Malawi.^11^. The majority of these had vessel obstruction at the capillary level and associated small zones of non-perfusion. The areas of non-perfusion matched areas of retinal whitening seen in malarial retinopathy, strongly supporting the hypothesis that microcirculatory obstruction and resulting hypoxia lead to retinal whitening. Approximately one-quarter of these patients also had larger occluded vessels (arterioles and venules) with larger associated zones of retinal non-perfusion.

If these patterns of non-perfusion, with extensive heterogeneity of microvascular obstruction, are mirrored in the brain then a model is suggested whereby there are multiple small zones of reduced perfusion resulting in gradients of tissue hypoxia. These may be partially or fully compensated by adjacent vasodilation and hyperperfusion. These multiple lacunae of hypoxia or ischaemia would be compatible with the absence of gross neurological deficits in most patients on recovery and also in keeping with the subtle neurocognitive deficits that are evident in African children years after an episode of CM.^12^, ^13^, ^14^

The retina provides a unique opportunity to observe the central nervous system vasculature directly and therefore to study cerebral vascular pathology directly. The only other technique that currently allows detailed, direct, relatively non-invasive observation of microcirculatory blood flow is orthogonal polarising spectroscopy (OPS). OPS has been used to demonstrate reduced microcirculatory flow in the rectal mucosa in adult patients with CM,^15^ although this technique has yet to be employed in children. The severity of flow obstruction in this study correlated with the severity of disease. The proportion of adults with capillary obstruction demonstrated by OPS was similar (67%) to that demonstrated in children by fluorescein angiography (76%).^11^

The other major hypothesis to explain the pathophysiology of CM is the local or systemic release of inflammatory mediators such as cytokines and nitric oxide.^16^ The evidence from the eye goes against this. Retinal whitening is seen more in watershed zones and not along vessels, as would be expected if a causal substance was ‘leaking’ from the blood into the surrounding tissues, whereas the former points more towards a perfusion deficit.

Studies of the integrity of the blood–brain barrier (BBB) in CM have relied on global measures of cerebral spinal fluid composition or on autopsy specimens in those that have died. These have been equivocal, showing minor increases in BBB permeability, and have not demonstrated a breakdown in the BBB to a degree that would account for the degree of brain swelling. Magnetic resonance imaging suggests the brain is congested with blood, and not full of water.^17^ The retina has a blood–retinal barrier (BRB) that is structurally and functionally the same as the brain, and its integrity is tested by fluorescein angiography. Although BRB breakdown occurred in 44% of children with CM, it only affected limited portions of a few vessels and to a minor degree.^11^ It was seen adjacent to ischaemic zones or in areas that had not been perfused but later recovered. It did not occur in discoloured vessels or vessels narrowed by sequestration. This suggests that breakdown of the blood–tissue barrier is a non-specific response to severe disease, possibly as a result of local hypoxia compounded by endothelial dysfunction.

## 4 Sequelae

Although patients with CM can have prolonged deep coma, neurological sequelae in adults are surprisingly rare, at approximately 1%.^18^ This may be because of the redundancy of the cerebral microvasculature or redundancy in the cerebral cortex, which can accommodate small ‘silent’ infarcts. There is substantial heterogeneity of vascular obstruction, with patent capillaries adjacent to obstructed ones as shown by OPS.^15^ In children, neurological sequelae are more frequent; approximately 12% at the time of discharge, although the majority resolve.^19^ If microcirculatory flow dysfunction is largely responsible for malarial retinopathy then it begs the question, is vision affected? In a study of 162 children with severe malaria, 143 of whom had cerebral disease, visual acuity at 1 month in the half who attended follow-up showed no correlation with the severity of retinopathy seen during admission.^20^ Methodological difficulties, including the use of a variety of visual assessment tools to accommodate this heterogeneous group of children in terms of age and patient co-operation, language and culture, may well have reduced the robustness of their findings. In addition, acuity was not tested acutely; the first measurement was at 30 days, allowing any temporary effects on acuity to resolve before testing. It remains unanswered whether retinal whitening and non-perfusion at the fovea affect vision acutely, and whether peripheral non-perfusion has a temporary or permanent affect on visual fields.

## 5 Adults and children

There are only three studies of retinopathy in severe malaria in adults.^21^, ^22^, ^23^, ^24^. An Indian study included 214 patients with CM of whom only 34.1% had retinopathy, with only disk pallor being significantly associated with increased mortality (*P *< 0.05).^21^, ^22^ The relative frequencies of retinal changes reported in this study differed significantly from those reported elsewhere. The second study was done in a mixed group of adults and children in Thailand, of which 144 patients had CM.^23^ It focused on retinal haemorrhages, which were found in 14.6% of patients, there being very few other retinal abnormalities found. Both studies used predominantly direct ophthalmoscopy and did not state the degree of expertise of the examiner. A large proportion of retinal changes in malaria are in the peripheral retina^4^ and the use of direct ophthalmoscopy by non-experts may account for the low incidences found. Neither study reported changes in retinopathy over time other than to say ‘recovery was complete’ in survivors. A study using film-based retinal angiography in adults to investigate capillary permeability showed some areas of capillary non-perfusion in the retina, similar to the recent findings in children.^24^ It is likely that the technology in this older study would not have been sufficient to demonstrate all the pathology demonstrated in children with digital angiography and a modern fundus camera.

Approximately one-third of patients with CM die during the acute phase of the disease. Currently there is debate whether the pathophysiological mechanisms leading to cerebral and severe malaria differ between adults and children. Pathology studies in adults show a clear correlation between the extent of sequestration in the brain vasculature and coma.^25^ In adult CM, platelets and white cells are usually not seen in sequestered segments, whereas in children in addition to sequestration there is some intravascular accumulation of mononuclear leukocytes and platelets as well as the presence of fibrin strands.^6^ It has not been established whether retinal pathology differs between adults and children.

## 6 Future research

The ability to visualise sequestration and its effects on neurological tissue in vivo raises the possibility that this could be used as a disease measure for investigative and interventional trials. Until now the only reliable indicators of efficacy of new agents have been an effect on death rates or neurological sequelae. These remain the ultimate measures, but it may be possible to study more upstream events and earlier effects. For instance, levamisole is an anthelminthic that is currently under evaluation as a novel adjunctive therapy for CM in adults. It is thought to reduce sequestration of parasitised erythrocytes by blocking their binding to vascular endothelial CD36.^26^ In a pilot study of adult patients with CM treated with quinine, levamisole increased peripheral blood parasitaemia and prevented sequestration of early and mid-trophozoite parasites.^27^ A larger study of patients with CM treated with artesunate is now underway. It remains to be seen whether these apparently beneficial effects of levamisole are clinically significant and whether other related medications have similar or perhaps greater effects. Malarial retinopathy may be a sensitive pharmacodynamic endpoint for the evaluation of such novel therapies.

## 7 Conclusion

It is likely that the retinopathy seen in severe malaria and malarial coma are manifestations of the same pathophysiological mechanisms acting at related anatomical sites. To date, no technique has allowed direct examination of the small blood vessels of the brain, although examination of a number of surrogate markers has led to the conclusion that obstruction of microcirculatory flow is an important contributor to CM. Retinal blood vessels are identical to those in the cerebrum and can be examined relatively easily. They provide a unique opportunity to increase our understanding of this still enigmatic disease. Recent clinical and pathological investigations suggest that multiple foci of capillary obstruction are important in the pathophysiology of severe malaria, and that breakdown of the BBB is a limited and secondary phenomenon. This improved understanding provides a platform for evaluation of better treatment strategies for CM.

## Funding

Mahidol Oxford Tropical Medicine Research Programme is funded by the Wellcome Trust of Great Britain.

## Conflicts of interest

None declared.

## Ethical approval

Not required
